# ﻿*Gastrochilus jiuzhaigouensis* (Orchidaceae, Epidendroideae), a new epiphytic orchid from Jiuzhaigou World Heritage Site, China

**DOI:** 10.3897/phytokeys.265.159138

**Published:** 2025-10-14

**Authors:** Da-Lin Zhu, Jun-Yi Zhang, Yue Zhang, Min Liao, Hai He, Ke Chen, Bo Xu

**Affiliations:** 1 College of Life Science and Agri-forestry, Engineering Research Center of Biomass Materials of Ministry of Education, Southwest University of Science and Technology, Mianyang 621010, China Southwest University of Science and Technology Mianyang China; 2 Mountain Ecological Restoration and Biodiversity Conservation Key Laboratory of Sichuan Province, Chengdu Institute of Biology, Chinese Academy of Sciences, Chengdu 610213, China Chinese Academy of Sciences Chengdu China; 3 College of Life Sciences, Sichuan University, Chengdu 610064, China Sichuan University Chengdu China; 4 University of Chinese Academy of Sciences, Beijing 10049, China University of Chinese Academy of Sciences Beijing China; 5 Jiuzhaigou National Nature Reserve Administrative Bureau, Aba Tibetan and Qiang Autonomous Prefecture 623402, Jiuzhaigou, China Jiuzhaigou National Nature Reserve Administrative Bureau Jiuzhaigou China; 6 College of Life Sciences, Chongqing Normal University, Chongqing 401331, China Chongqing Normal University Chongqing China

**Keywords:** *
Gastrochilus
*, Jiuzhaigou, new species, Orchidaceae, phylogeny

## Abstract

*Gastrochilus jiuzhaigouensis* (Orchidaceae), a new orchid species epiphytic on tree branches on the bank of an alpine lake at the famous Jiuzhaigou National Park in China, is described and illustrated. It differs from its morphologically similar species, *G. bernhardtianus*, by its shorter stems, obliquely linear-lanceolate leaves, reniform and revolute epichile without ornamentation, and the absence of a middle ridge at central callus of the epichile. To test its species status, sequences of the nuclear ribosomal internal transcribed spacer (nrITS) and four chloroplast DNA markers (*matK*, *psbA-trnH*, *psbM-trnD*, and *trnL-F*) were newly generated. Molecular phylogenetic analyses including 53 accessions of *Gastrochilus* species resolved it within the same clade as *G. bernhardtianus*, but with distinct boundary. Both morphological differences and phylogenetic evidence supported it a species new to science.

## ﻿Introduction

The vandoid genus *Gastrochilus* D.Don (Orchidaceae, Epidendroideae; Don 1825) includes about 80 epiphytic species distributed in tropical and subtropical Asia (Don 1825; Tsi 1996, 1999; Liu et al. 2019; Govaerts et al. 2021; Zhou et al. 2021; Zhang et al. 2022; Ya et al. 2023; Zhang et al. 2024a, b; Xiong et al. 2025). The generic morphological characters were mainly defined by the enlarged and saccate hypochile, and two sub-globose pollinia borne on a slender stipe (Tsi 1996; Pridgeon et al. 2014; Liao et al. 2022; Zhang et al. 2024a). Incorporated with the recently molecular phylogenetic resolved six clades, it was subdivided into six sections using characters of leaf shape and length, margin of the epichile, and indumentum on the surface of epichile (Zhang et al. 2024a). However, the diversity of this genus is far from well documented, with continuous new discoveries, especially in the Hengduan Mountains (Liu et al. 2019; Rao et al. 2019; Wu et al. 2019; Chen et al. 2022; Liao et al. 2022; Zhang et al. 2022; Nguyen et al. 2022; Lee et al. 2023; Liu et al. 2023; Ya et al. 2023; Zhang et al. 2024a, b; Zhou et al. 2024).

The Jiuzhaigou World Heritage Site is part of the Min Mountains of the Hengduan Mountains. The Min Mountains are one of Southwest China’s most important biodiversity hotspots (Tang et al. 2006). In April 2024, during a field trip in Jiuzhaigou National Park (part of the Jiuzhaigou World Heritage Site in Jiuzhaigou County, Sichuan Province, China), we encountered a unique *Gastrochilus* species with distichously alternate linear-lanceolate leaves and small flowers with a reniform epichile. It could be assigned to *G. sect. Microphylli* (Benth. & Hook.f.) Z.H.Tsi (Tsi 1996; Zhang et al. 2024a) based on its general leaf morphology and floral characters. However, it does not match any known species in the Chinese floras (e.g., Tsi 1999; Chen et al. 2009) and superficially resembles *G. bernhardtianus* J.D.Ya & D.Z.Li, recently described from Lijiang, Northwest Yunnan (Ya et al. 2023). We hereafter suppose it to be a new species and have named it *G. jiuzhaigouensis*. To test its systematic position and to determine whether this plant is sufficiently distinct from other known species of *Gastrochilus* to merit a status of new species, we conducted morphological comparisons and molecular phylogenetic analyses.

## ﻿Materials and methods

### ﻿Morphological analyses

Herbarium specimens and silica-gel-dried leaves of this new *Gastrochilus* plant were collected in the field at Wuhuahai (the Colorful Lake) in Jiuzhaigou National Park, Jiuzhaigou County, Sichuan Province, Southwest China. Morphological measurements were based on both living plants and dried herbarium specimens deposited at CDBI (herbarium acronyms follow Thiers 2025). The terminology proposed by Beentje (2016) was followed. The sequence of the taxonomic description was referred to the style of the Flora of China (Chen et al. 2009). Detailed morphological characters of *G. bernhardtianus*, *G. balangshanensis* Jun Y. Zhang, Bo Xu & Yue H. Cheng (Zhang et al. 2024b), and this species were compared by reviewing type materials at CDBI and KUN (by Jun Yi Zhang and Min Liao).

### ﻿DNA extraction, amplification, and sequencing

Total DNA was extracted exclusively from silica-gel-dried leaves using a Plant DNA Isolation Kit (Cat. No. DE-06111). Based on the phylogenetic studies of *Gastrochilus* by Liu et al. (2019) and Zhang et al. (2024a, b), we applied the same primers to amplify the nuclear ribosomal internal transcribed spacer (nrITS) and four chloroplast DNA fragments (*matK*, *psbA-trnH*, *psbM-trnD*, and *trnL-F*) through polymerase chain reaction (PCR). All DNA samples were sequenced by TSINGKE Biotech Co. Ltd. (Chengdu, China). The final manually corrected sequences were submitted to GenBank (Table A1).

### ﻿Phylogenetic analyses

The sequences of 58 species included in the molecular phylogenetic analysis, originally published in Liu et al. (2019), Ya et al. (2023), Zhang et al. (2024a, b), and Xiong et al. (2025), were retrieved from GenBank, except for those obtained from two individuals of the novelty, which were newly generated in this study. Detailed information concerning the DNA markers, sampled taxa, voucher collections, and GenBank accession numbers is listed in Table A1.

All sequences were edited using Sequencher v4.1.4 (Gene Codes, Ann Arbor, Michigan, USA) and aligned using MAFFT v7.475 (Katoh and Standley 2013) with default parameters. Phylogenetic analyses were performed based on the combined dataset of nuclear ribosomal internal transcribed spacer (nrITS) and the four chloroplast DNA fragments, after checking for congruence, with six Aeridinae species in the genera *Luisia* Gaudich., *Saccolabium* Blume, *Holcoglossum* Schltr., and *Pomatocalpa* Breda as outgroups (Liu et al. 2019; Zhang et al. 2024a). The nucleotide substitution model for the data matrix was estimated using jModelTest v2.1.6 (Posada 2008), and the best-fit evolutionary model (GTR+F+I+G4) was selected using the corrected Akaike Information Criterion (AICc). Maximum likelihood (ML) and Bayesian inference (BI) methods were employed for phylogenetic tree reconstruction. The BI analysis was conducted using MrBayes v3.2.7a (Ronquist and Huelsenbeck 2003) with two separate Markov chain Monte Carlo (MCMC) runs (20,000,000 generations, sampled every 1,000 generations). The first 25% of the trees were discarded as burn-in, and the remaining trees were used to generate a majority-rule consensus tree. The ML analysis was performed using IQ-TREE v1.4.2 (Nguyen et al. 2014), with branch support estimated using 2,000 replicates. The resulting phylogenetic trees were visualized using Chiplot (Xie et al. 2023).

## ﻿Results

**Figure 1.**
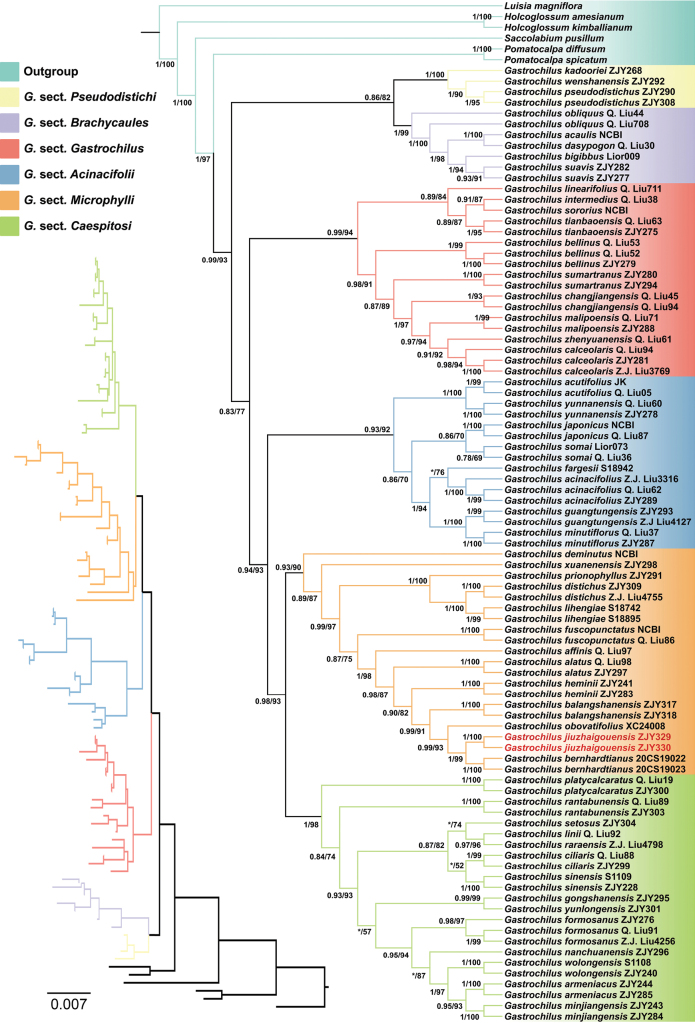
Maximum likelihood phylogenetic tree of *Gastrochilus*, including 53 taxa, based on the combined nrITS and four-plastid (*mat*K, *psb*A–*trn*H, *psb*M–*trn*D, and *trn*L–F) markers. Values before the slash indicate Bayesian posterior probabilities, and numbers after the slash indicate ML bootstrap supports for major lineages. An asterisk (*) indicates that a node is not supported in the analysis. The two accessions of the inferred new species are highlighted in red, and the colors of the terminal nodes correspond to the six sections of *Gastrochilus* defined in Zhang et al. (2024a).

The aligned nrITS dataset is 684 nucleotides long with 197 variable sites, and the combined four plastid markers dataset included 3,448 nucleotides in length with 212 variable sites, consists of 805 bp for *matK*, 676 bp for *psbA-trnH*, 943 bp for *psbM-trnD*, and 1,024 bp for *trnL-F*. Consistent with previous studies, *Gastrochilus* is resolved as a strongly supported monophyly with high posterior probabilities (PP) and bootstrap probabilities (BP). It also agrees with the previous subdivision within the genus in that the clades corresponding to the six sections are well supported (Fig. 1). The two accessions of *G. jiuzhaigouensis* cluster in one lineage (BI/ML = 1/100; Fig. 1), which is a sister lineage to the two accessions of *G. bernhardtianus* J.D.Ya & D.Z.Li (BI/ML = 1/99; Fig. 1). These two species are more closely related to *G. obovatifolius* C. Xiong, X.Y. Fu & S.R. Yi and *G. balangshanensis*, among others that can be grouped within *G. sect. Microphylli* (Fig. 1).

*Gastrochilus bernhardtianus* shows similarity to *G. jiuzhaigouensis* in plant size, distichous leaves, and gross floral characters, but they mainly differ in stem length and lip morphology. The general morphology of *G. balangshanensis* and *G. obovatifolius* is more distinguishable from *G. jiuzhaigouensis*, and their detailed morphological comparisons are summarized in Table 1.

**Table 1.** Morphological comparisons of *Gastrochilus jiuzhaigouensis* with three related species of G. sect. Microphylli.

Character	G. jiuzhaigouensis	G. bernhardtianus	G. balangshanensis	G. obovatifolius
Stem length	0.5–1.2 cm	ca. 5.0 cm	1.5–3.5 cm	ca. 5.0 cm
Leaves	obliquely linear-lanceolate, 1.2–2.4 × 0.3–0.6 cm	oblong-lanceolate,1.8–2.5 × 0.4–0.7 cm	nearly elliptic, 0.9–1.5 × 0.4–0.8 cm	obovate, 1.4–1.6 × 0.6–0.8 cm
Dorsal sepal	4.8–6.0 × 3.6–4.2 mm	ca. 5.2 × 3.4 mm	ca. 5.6–6.4 × 4.8–5.2 mm	ca. 5.0 × 4.0 mm
Lateral sepals	oblong, 5.0–6.8 × 3.0–4.0 mm	narrowly ovate, 5.5 × 2.8 mm	oblong, 5.0–5.8 × 4.0–4.4 mm	elliptic, slightly oblique, ca. 5.0 × 3.0 mm
Petals	obovate, 4.0–5.8 × 3.0–4.0 mm	narrowly oblong, ca. 5.2 × 2.7 mm	oblong, 5.0–5.8 × 4.0–4.4 mm	oblong, ca. 6.0 × 3.0 mm
Epichile (lip lamina)	reniform, revolute, 8.0–10.0 × 4.2–5.5 mm, central thickened purple-yellow thickening smooth without ornamentation	transversely oblong, spread, ca. 8.0 × 2.8 mm, central thickened yellow-green mat with 2 conic calli near its base	reniform, revolute, 10.0–12.0 × 5.5–6.5 mm, central thickened purple-red mat with two inconspicuous ridges	reniform, revolute, 10.0–12.0 × 6.0–8.0 mm, revolute, margin erose, median patch dark purple with 2 low ridges
Hypochile	sub-hemispherical, 4.0–5.0 × 3.8–5.4 mm, dorsally compressed, obtuse-rounded at the apex	subconical, ca. 5.1 × 3.8 mm, dorsally compressed splits into two conical sacs at the apex	sub-hemispherical, 6.0–8.0 × 5.8–7.5 mm, dorsally compressed, obtuse-rounded at the apex	sub-hemispherical, 4.0–4.5 × 4.0–4.2 mm, dorsally compressed, slightly bent outward, obtuse at the apex
Distribution	Jiuzhaigou, North Sichuan	Yulong, NE. Yunnan	Wenchuan, Central Sichuan	Chengkou, NE. Chongqing

## ﻿Discussion

Our phylogenetic analysis and morphological comparison with similar species support that *Gastrochilus jiuzhaigouensis* is a distinct species meriting description. Notably, we found plants of this species only as epiphytes on dieback branches of four trees immediately on the bank of the Colorful Lake (Fig. 3C–E). Falling of these branches may destroy its habitat. However, because the Colorful Lake is located in a deep valley, a more extensive investigation is necessary to assess its population size and conservation status. Since the Colorful Lake attracts thousands of sightseeing visitors daily, the introduction of this new epiphytic orchid will arouse public awareness of the protection of species diversity in Jiuzhaigou National Park. Discovery of this species increases the number of species in *G. sect. Microphylli* to 16 species, and it also represents the northernmost extension of the generic distribution in China (Chen et al. 2009; Zhang et al. 2024b; Xiong et al. 2025; Fig. 4).

Interestingly, the recently added four species—*G. bernhardtianus*, *G. obovatifolius*, *G. balangshanensis*, and *G. heminii* M.Liao, Bo Xu & Yue H.Cheng (Liao et al. 2022)—together with this new species were all resolved in one clade. This grouping was also reached by Xiong et al. (2025), using the accessions of the former four species, and by Ya et al. (2023), including accessions of only *G. bernhardtianus* and *G. heminii*. This implies that they may all radiate from an ancestral lineage. Concerning the phylogenetic relationship among these five species, our resolution showed higher support at each node by adding the accession of *G. jiuzhaigouensis*, compared with the results of Xiong et al. (2025). When revised circumscriptions were made for the subdivisions of *Gastrochilus*, *G. sect. Microphylli* was defined as having leaves narrow and less than 5 cm in length (Zhang et al. 2024a). The leaves of *G. jiuzhaigouensis* meet this criterion, showing even narrower linear-lanceolate outlines. It is worth noting that *G. balangshanensis* and *G. obovatifolius* extend leaf variation in this section to oblong and obovate forms (Zhang et al. 2024b; Xiong et al. 2025; Table 1).

With respect to geographical distribution (Fig. 4), the most recently discovered and closely related species are assumed to be either endemic to the Hengduan Mountains in Southwest China (Liao et al. 2022; Ya et al. 2023; Zhang et al. 2024b) or to the Qinling–Dabashan Mountains (Xiong et al. 2025). In southwestern China, mountainous areas around the Sichuan Basin (Fig. 4) could provide suitable habitats for these epiphytic orchids. Extensive investigations may override the apparently disjunct distributions of these species, especially the phylogenetically close ones. Concerning the recently described oblong or obovate leaves of species in *G. sect. Microphylli*, a species endemic to Nanchuan, Chongqing (cited as “Sichuan” before July 1997), also has oblong or ovate leaves (Tsi 1996, 1999; Chen et al. 2009). Though there are several gatherings deposited at PE and IMC, all were collected from one locality, with elevations variably documented from 750 m to 1,200 m. In agreement with previous studies (e.g., Ya et al. 2023; Zhang et al. 2024a), our present phylogenetic analysis (Fig. 1) resolved it in a clade composed of other species in *G. sect. Caespitosi* Z.H.Tsi (Tsi 1996), though it was originally ascribed to *G. sect. Microphylli* (Tsi 1996, 1999). The flower color, hairy epichile, and phenology (flowering in December), among other characters, are very different from *G. jiuzhaigouensis* and its closely related species. However, future studies may help to close the distributional gaps of *Gastrochilus* species, and increased sampling might also blur the presently resolved sectional boundaries.

### ﻿Taxonomic treatment

#### ﻿ Gastrochilus jiuzhaigouensis

Taxon classificationPlantaeAsparagalesOrchidaceae

Jun Y.Zhang, D.L.Zhu & Y.Zhang sp. nov.

30AC946F-6AC7-5E7E-90A0-077F1AD0670F

urn:lsid:ipni.org:names:77370514-1

Figs 2D, E, 3

##### Type.

China • Sichuan: Jiuzhaigou, Wuhuahai (the Colorful Lake), lake bank trees at border of subalpine mixed coniferous forest, on dieback tree branches, elev. ca. 2,478 m, in flower, 23 April 2024, *Jun-Yi Zhang & Da-Lin Zhu ZJY195* (Holotype CDBI!; Isotype CDBI!).

##### Diagnosis.

It is most similar to *G. bernhardtianus*, but it can be distinguished by its shorter stem (0.5–1.2 vs. ca. 5.0 cm), obliquely linear-lanceolate leaves (vs. oblong-lanceolate leaves), reniform and revolute epichile (vs. transversely oblong and spread) without ornamentation (vs. two conic calli near its base), and absence of middle ridge (vs. distinctly ridged) at the middle of central callus on the epichile.

**Figure 2.**
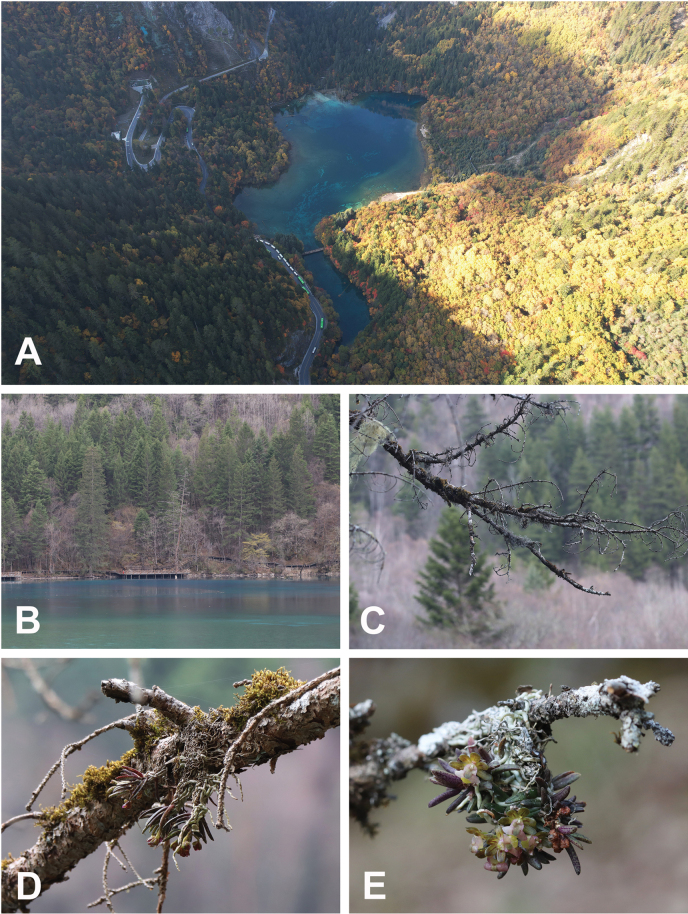
Habitat and habit of *Gastrochilus jiuzhaigouensis* in situ. A, B. Habitat; C–E. Flowering plants of *G. Jiuzhaigouensis* growing on tree branches. [Images A–E. Photographed by Da-Lin Zhu].

##### Description.

Miniature branch epiphyte, pendent, 2.0–4.0 cm tall. Roots vermiform, 3.0–5.0 cm long, ca. 1.2 mm in diameter. Stem unbranched, 0.5–1.2 cm long, ca. 1.2 mm in diameter. Leaves thickly leathery (with fleshy mesophyll), tinged with purplish-red spots, closely arranged, distichous, alternate; blade obliquely linear-lanceolate, 1.2–2.4 × 0.3–0.6 cm, base slightly asymmetry, apex acute and ended with one lobule, lobes setaceous. Inflorescence a 1- or 2-flowered on a short rachis; peduncle curved upward and thickened, 3.0–6.0 mm long, proximally covered with one sheath; floral bracts ovate-lanceolate, 0.6–1.0 mm long, apex acute; pedicel and ovary connate, 0.8–1.1 cm long. Flowers spreading, ca. 0.8 × 1.2 cm; sepals and petals yellow-green on both sides; dorsal sepals elliptic, 4.8–6.0 × 3.6–4.2 mm, apex obtuse; lateral sepals oblong, 5.0–6.8 × 3.0–4.0 mm, apex obtuse; petals obovate, 4.0–5.8 × 3.0–4.0 mm, apex obtuse, base narrowed; lip with a reniform epichile, revolute, purplish-red, 8.0–10.0 × 4.2–5.5 mm, margin entire, smooth and glabrous above, centra thickened purple-yellow without ornamentation; hypochile sub-hemispherical, yellowish-green, 4.0–5.0 × 3.8–5.4 mm, dorsally compressed, obtuse-rounded at the apex; column cylindrical, ca. 1.0 mm; anther cap galeate with two chambers, ca. 0.8 × 0.5 mm; rostellum bilobed; pollinarium ca. 0.3 mm long; pollinia 2, ca. 0.3 × 0.2 mm, yellow, subglobular, porate; stigma deeply sunken, inverted V-shaped, ca. 0.5 mm long, white; rostellum bilobed. Fruits not mature.

**Figure 3.**
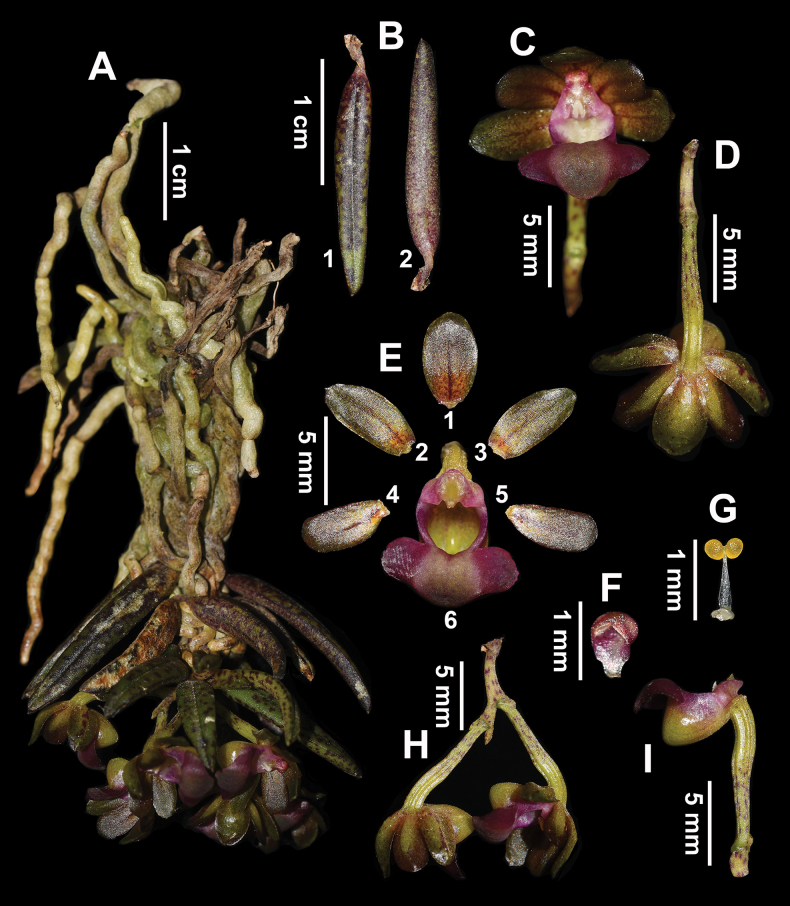
*Gastrochilus jiuzhaigouensis*. A. Flowering plant; B. Leaves (B1: front view, B2: abaxial view); C. Flowers, front view; D. Flowers, abaxial view; E. Dissection of a flower (E1: dorsal sepal; E2-3: lateral sepals; E4-5: petals; E6: labellum); F. Anther cap; G. Pollinarium with pollinia; H. Raceme, lateral view; I. Labellum, lateral view. [Images A–I. photographed by Jun-Yi Zhang].

##### Distribution and habitat.

**Figure 4.**
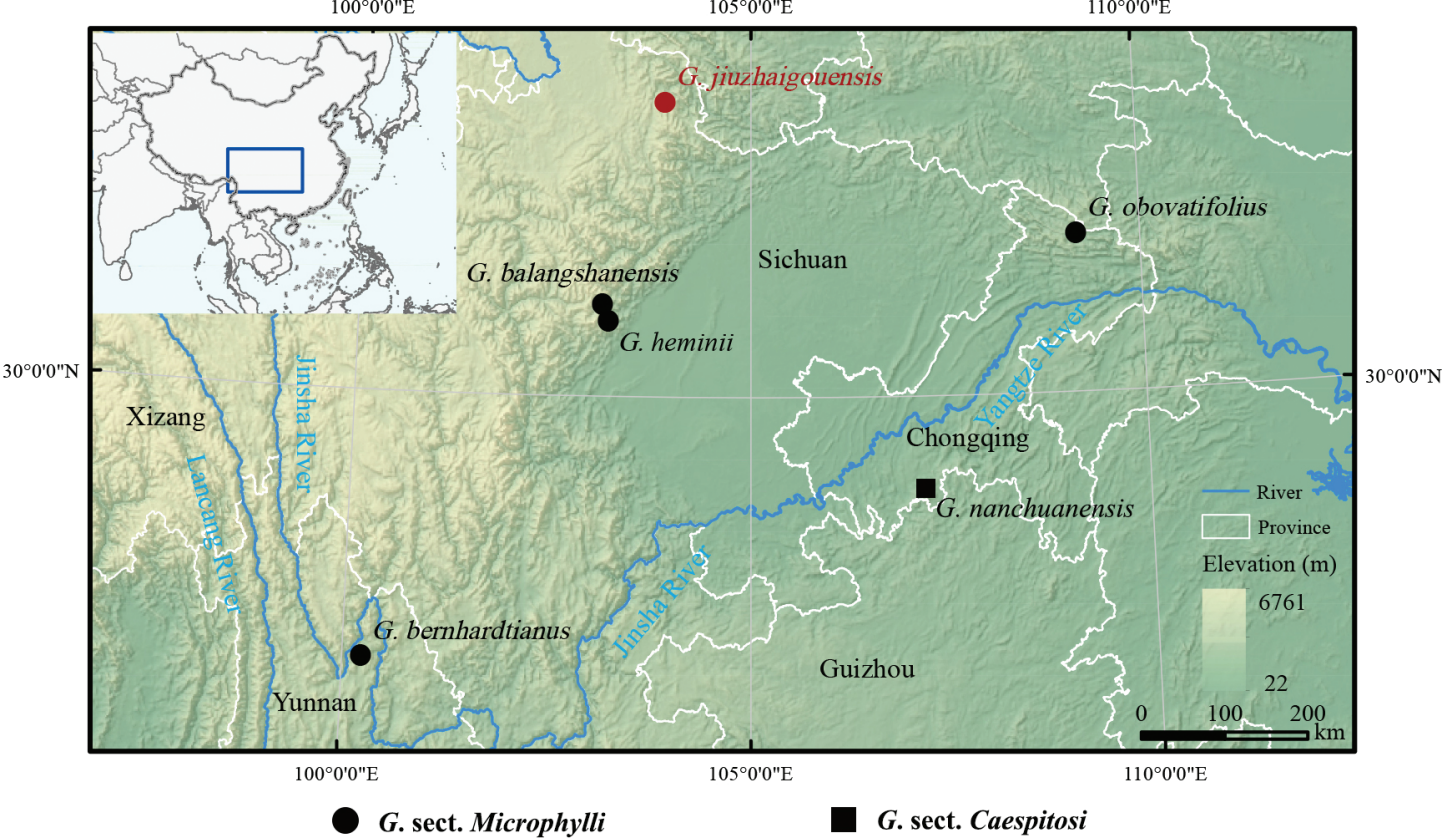
Distribution map of *Gastrochilus jiuzhaigouensis* and five related species in *Gastrochilus*; *G. jiuzhaigouensis, G. balangshanensis*, *G. bernhardtianus*, and *G. obovatifolius* are grouped in G. sect. Microphylli, and *G. nanchuanensis* in G. sect. Caespitosi.

*Gastrochilus jiuzhaigouensis* is currently known only from Wuhuahai, Jiuzhaigou County, Sichuan Province, Southwest China (Fig. 4). It is epiphytic on dieback tree branches on the bank of a lake of trees in the border of subalpine mixed coniferous and deciduous broadleaf forest dominated by *Pinus tabuliformis* Carrièreat at elevational range between 2400 and 2500 m. Other common accompanying broadleaf trees include Acer stachyophyllum subsp. betulifolium (Maxim.) P.C. De Jong, *Fraxinus chinensis* Roxb., *Prunus tomentosa* Thunb., and *Quercus mongolica* Fisch. ex Ledeb., among others. And in shrub layer *Berberis amurensis* Rupr. can reach a height of two to three meters.

##### Phenology.

Flowering from March to April.

##### Etymology.

Its specific epithet is taken from name of Jiuzhaigou World Heritage Site, where the type specimen of this new orchid is located. Jiuzhaigou is also the name of the famous Jiuzhaigou National Park and the county name in Northwest Sichuan. It is therefore the Chinese name that is proposed as *jiu zhai gou peng ju lan* (九寨沟盆距兰).

##### Additional specimens examined

**(paratypes).** China • Sichuan: Jiuzhaigou, Wuhuahai, on lake bank of broadleaf tree at the border of subalpine coniferous mixed forest, on tree branches, elev. ca. 2,478 m, in flower, 23 April 2024, *Jun-Yi Zhang & Da-Lin Zhu ZJY197* (CDBI!).

##### Other specimens examined.

***Gastrochilus bernhardtianus*.** China • Yunnan: Lijiang Prefecture, Yulong County, Yunshanping, elev. ca. 3308 m, in cold-temperate, evergreen conifer forest, in flower, 20 May 2020, *J.-D. Ya et al. 20CS19022* (holotype KUN).

***Gastrochilus balangshanensis*.** China • Sichuan: Wenchuan, Balangshan, Yinchangou, mixed coniferous forest, on tree branches, elev. ca. 2,260 m, in flower, 19 April 2023, *Jun-Yi Zhang & Yue-Hong Cheng ZJY185* (holotype: CDBI!); *ibid. loc.*, mixed coniferous forest, on tree trunk, elev. ca. 2300 m, in flower, 19 April 2023, *Jun-Yi Zhang & Yue-Hong Cheng ZJY186* (CDBI!); *ibid. loc.*, mixed coniferous forest, on tree branch, elev. ca. 2315 m, in fruit, 22 July 2024, *Jun-Yi Zhang & Yue-Hong Cheng ZJY204* (CDBI!).

## Supplementary Material

XML Treatment for Gastrochilus jiuzhaigouensis

## ﻿Additional information

### Use of AI

No use of AI was reported.

### Author ORCIDs

Da-Lin Zhu https://orcid.org/0009-0009-4860-494X

Jun-Yi Zhang https://orcid.org/0000-0001-8325-5954

Min Liao https://orcid.org/0000-0001-6777-8579

Hai He https://orcid.org/0000-0001-5362-9177

### Data availability

All of the data that support the findings of this study are available in the main text.

## ﻿Appendix 1

**Table A1.** Voucher information and GenBank accession numbers of DNA sequences of *Gastrochilus* used in phylogenetic inference.

Taxa	Voucher	Location	nrITS	matK	psbA–trnH	trnL–F	psbM–trnD
*G. acaulis* (Lindl.) Kuntze	—	—	KM583455	KM583465	—	—	—
*G. acinacifolius* Z.H.Tsi	*Z. J. Liu 3316*	CHINA	KJ733412	KJ733569	KJ733492	KJ733649	—
*G. acinacifolius* Z.H.Tsi	*Q. Liu 62*	CHINA: Hainan (Baomeiling)	MK357118	MK357138	MK357160	MK357208	MK357216
*G. acinacifolius* Z.H.Tsi	*ZJY289*	CHINA: Yunnan (Malipo)	OQ566796	OQ575562	OQ575590	OQ575530	OQ575619
*G. acutifolius* (Lindl.) Kuntze	*Q. Liu 05*	CHINA: Yunnan (Jingmai)	—	MK357140	MK357162	—	MK357230
*G. acutifolius* (Lindl.) Kuntze	*JK-DEBCR-mat-31* (JK)	—	MW475270	MW433889	—	—	—
*G. affinis* (King & Pantl.) Schltr.	*Q. Liu 97*	CHINA: Yunnan (Fugong)	—	MK357141	MK357163	—	MK357141
*G. alatus* X.H.Jin & S.C.Chen	*Q. Liu 98*	CHINA: Yunnan (Bingzhongluo)	—	—	—	—	MK357228
*G. alatus* X.H.Jin & S.C.Chen	*ZJY297*	CHINA: Yunnan	—	OQ575563	OQ575591	OQ575531	OQ575620
*G. armeniacus* Jun Y.Zhang, B.Xu & Yue H.Cheng	*ZJY244*	CHINA: Sichuan (Wenchuan)	OP348889	OP373113	OP373119	OP373128	OP373123
*G. armeniacus* Jun Y.Zhang, B.Xu & Yue H.Cheng	*ZJY285*	CHINA: Sichuan (Wenchuan)	OP348888	OP373114	OP373118	OP373127	OP373124
*G. balangshanensis* Jun Y.Zhang, B.Xu & Yue H.Cheng	*ZJY317*	CHINA: Sichuan (Wenchuan)	PP960151	PP944493	PP944495	PP944499	PP944497
*G. balangshanensis* Jun Y.Zhang, B.Xu & Yue H.Cheng	*ZJY318*	CHINA: Sichuan (Wenchuan)	PP960152	PP944494	PP944496	PP944500	PP944498
*G. bellinus* (Rolfe. F.) Kuntze	*Q. Liu 52*	CHINA: Yunnan (Lancang)	KY966597	KY966884	—	—	—
*G. bellinus* (Rolfe. F.) Kuntze	*Q. Liu 53*	CHINA: Yunnan (Mengsong)	MK357123	MK357142	MK357164	MK357202	MK357241
*G. bellinus* (Rolfe. F.) Kuntze	*ZJY279*	CHINA: Yunnan (Mengla)	OQ566799	OQ575564	OQ575592	OQ575534	OQ575621
*G. bernhardtianus* J.D.Ya & D.Z.Li	*20CS19022*	CHINA: Yunnan (Lijiang)	OR073404	20CS19022	20CS19022	20CS19022	20CS19022
*G. bernhardtianus* J.D.Ya & D.Z.Li	*20CS19023*	CHINA: Yunnan (Lijiang)	OR073405	20CS19023	20CS19023	20CS19023	20CS19023
*G. bigibbus* (Rchb. f. ex Hook. f.) Kuntze	*Li 009*	—	—	MN124439	MN124439	MN124439	MN124439
*G. calceolaris* D.Don	*Z. J. Liu 3769*	CHINA	KF545874	KF545885	KF545865	—	KF545896
*G. calceolaris* D.Don	*Q. Liu 94*	CHINA: Yunnan (Jingdong)	MK357126	MK357144	MK357169	MK357205	MK357233
*G. calceolaris* D.Don	*ZJY281*	CHINA: Yunnan (Malipo)	—	OQ575565	OQ575593	OQ575535	OQ575622
*G. changjiangensis* Q.Liu & M.Z.Huang	*Q. Liu 45*	CHINA: Hainan (Changjiang)	MK357124	—	MK357166	—	MK357236
*G. ciliaris* F.Maekawa	*Q. Liu 88*	CHINA: Nursery of Management Office of Yushan Scenic Area, Fuzhou	—	MK357148	MK357173	—	MK357225
*G. ciliaris* F.Maekawa	*ZJY299*	CHINA: Taiwan	—	OQ575566	OQ575594	—	OQ575623
*G. dasypogon* (Lindl.) Kuntze	*Q. Liu 30*	CHINA: Hainan (Baomeiling)	MK357129	MK357149	MK357181	MK357197	MK357219
*G. deminutus* J.M.H.Shaw	—	—	KY966600	KY966887	—	—	—
*G. distichus* (Lindl.) Kuntze	*Z. J. Liu 4755*	CHINA	KJ733414	KJ733571	KJ733494	KJ733651	—
*G. distichus* (Lindl.) Kuntze	*ZJY309*	CHINA: Yunnan (Malipo)	OQ566800	OQ575567	OQ575595	OQ575536	—
*G. fargesii* (Kraenzl.) Schltr.	*20HT3548*	CHINA: Chongqing	OR073405	S18942	S18942	S18942	S18942
*G. formosanus* (Hayata) Hayata	*Z. J. Liu 4256*	CHINA	KJ733416	KJ733573	KJ733495	KJ733653	—
*G. formosanus* (Hayata) Hayata	*Q. Liu 91*	CHINA: Yunnan (Gongshan)	—	—	MK357174	—	MK357226
*G. formosanus* (Hayata) Hayata	*ZJY276*	CHINA: Hubei (Enshi)	OQ566801	OQ575568	OQ575596	OQ575537	OQ575624
*G. fuscopunctatus* (Hayata) Hayata	*Q. Liu 86*	CHINA: Taiwan (Hualian)	—	MK357150	MK357171	MK357192	MK357231
*G. fuscopunctatus* (Hayata) Hayata*	—	—	—	KX871233	KX871233	KX871233	KX871233
*G. gongshanensis* Z.H.Tsi	*ZJY295*	CHINA: Yunnan (Malipo)	—	OQ575569	OQ575597	OQ575538	OQ575625
*G. guangtungensis* Z.H.Tsi	*Z. J. Liu 4127*	CHINA	KJ733417	KJ733574	KJ733496	KJ733654	—
*G. guangtungensis* Z.H.Tsi	*ZJY293*	CHINA: Yunnan	OQ566802	OQ575570	OQ575598	OQ575539	OQ575626
*G. heminii* M.Liao, B.Xu & Yue.H.Cheng	*ZJY241*	CHINA: Sichuan (Wenchuan)	ON286752	ON331126	ON331128	ON331130	ON331132
*G. heminii* M.Liao, B.Xu & Yue.H.Cheng	*ZJY283*	CHINA: Sichuan (Wenchuan)	ON286753	ON331127	ON331129	ON331131	ON331133
*G. intermedius* (Griff. ex Lindl.) Kuntze	*Q. Liu 38*	CHINA: Yunnan (Yinchang)	MK357121	MK357151	MK357172	MK357190	MK357213
*G. japonicus* (Makino) Schltr.	*Q. Liu 87*	CHINA: Taiwan (Pingdong)	KF545875	KF545886	KF545866	KF545897	—
*G. japonicus* (Makino) Schltr.	—	—	—	KX871236	KX871236	KX871236	KX871236
***G. jiuzhaigouensis Jun Y.Zhang, D.L.Zhu & Y.Zhang***	***ZJY329***	**CHINA: Sichuan (Jiuzhaigou)**	**PV643100**	**PV645752**	**PV645754**	**PV645758**	**PV645756**
***G. jiuzhaigouensis Jun Y.Zhang, D.L.Zhu & Y.Zhang***	***ZJY330***	**CHINA: Sichuan (Jiuzhaigou)**	**PV643101**	**PV645753**	**PV645755**	**PV645759**	**PV645757**
*G. kadooriei* Kumar et al.	*ZJY268*	CHINA: Yunnan (Malipo)	OQ566803	OQ575571	OQ575599	OQ575540	OQ575627
*G. lihengiae* J.D.Ya, H.Jiang & D.Z.Li	*22CS21828*	CHINA: Yunnan (Gongshan)	OR073408	S18742	S18742	S18742	S18742
*G. lihengiae* J.D.Ya, H.Jiang & D.Z.Li	*23CS24145*	CHINA: Yunnan (Gongshan)	OR073408	S18895	S18895	S18895	S18895
*G. linearifolius* Z.H.Tsi & Garay	*Q. Liu 711*	MYANMA: Hkakaborazi National Park	MK357133	MK357136	MK357187	MK357194	MK357229
*G. linii* Ormerod	*Q. Liu 92*	CHINA: Nursery of Management Office of Yushan Scenic Area, Fuzhou	—	MK357152	MK357176	MK357198	MK357224
*G. malipoensis* X.H.Jin & S.C.Chen	*Q. Liu 71*	CHINA: Yunnan (Nanwenghe)	—	MK357147	MK357177	MK357200	MK357235
*G. malipoensis* X.H.Jin & S.C.Chen	*ZJY288*	CHINA: Yunnan (Malipo)	OQ566804	OQ575572	OQ575600	OQ575541	OQ575628
*G. matsuran* (Makino) Schltr.	—	—	KT338700	—	—	—	—
*G. minjiangensis* Jun Y.Zhang, B.Xu & Yue H.Cheng	*ZJY243*	CHINA: Sichuan (Wenchuan)	OP348887	OP373112	OP373117	OP373126	OP373122
*G. minjiangensis* Jun Y.Zhang, B.Xu & Yue H.Cheng	*ZJY284*	CHINA: Sichuan (Wenchuan)	OQ566806	—	—	OQ575542	—
*G. minutiflorus* Aver.	*Q. Liu 37*	CHINA: Yunnan (Malipo)	—	MK357153	MK357179	—	MK357215
*G. minutiflorus* Aver.	*ZJY287*	CHINA: Yunnan (Malipo)	OQ566807	OQ575573	OQ575601	OQ575544	OQ575629
*G. nanchuanensis* Z.H.Tsi	*ZJY296*	CHINA: Chongqing (Nanchuan)	OQ566808	OQ575574	OQ575602	OQ575545	OQ575630
G. obliquus var. obliquus (Lindl.) Kuntze	*Q. Liu 708*	MYANMA: Hponkanrazi Wildlife Sanctuary	MK357131	MK357137	KJ733498	KJ733656	MK357211
G. obliquus var. obliquus (Lindl.) Kuntze	*Q. Liu 44*	CHINA: Yunnan (Yinchang)	MK357130	MK357154	MK357182	MK357195	MK357218
*G. obovatifolius* C.Xiong, X.Y.Fu & S.R.Yi	*XC24008*	CHINA: Chongqing (Chengkou)	PP949380	PP942372	PP942372	PP942372	PP942372
*G. suavis* Seidenf.	*ZJY277*	CHINA: Yunnan	OQ566809	OQ575575	OQ575603	OQ575546	OQ575631
*G. suavis* Seidenf.	*ZJY282*	CHINA: Yunnan (Maguan)	OQ566810	OQ575576	OQ575604	OQ575547	OQ575632
*G. platycalcaratus* (Rolfe) Schltr.	*Q. Liu 19*	CHINA: Yunnan (Yinchang)	MK357122	—	MK357175	—	MK357222
*G. platycalcaratus* (Rolfe) Schltr.	*ZJY300*	CHINA: Yunnan (Mengla)	OQ566811	—	OQ575605	OQ575548	OQ575633
*G. prionophyllus* H.Jiang, D.P.Ye & Q.Liu	*ZJY291*	CHINA: Yunnan	OQ566812	OQ575577	OQ575606	OQ575549	OQ575634
*G. pseudodistichus* (King & Pantl.) Schltr.	*ZJY308*	CHINA: Yunnan (Malipo)	OQ566813	OQ575578	OQ575607	OQ575550	OQ575635
*G. pseudodistichus* (King & Pantl.) Schltr.	*ZJY290*	CHINA: Yunnan (Maguan)	OQ566814	OQ575579	OQ575608	OQ575551	OQ575636
*G. rantabunensis* S.Chow ex T.P.Lin	*Q. Liu 89*	CHINA: Taiwan (Taipei)	—	MK357155	MK357184	MK357193	MK357223
*G. rantabunensis* S.Chow ex T.P.Lin	*ZJY303*	CHINA: Taiwan	OQ566815	OQ575580	OQ575609	OQ575552	OQ575637
*G. raraensis* Fukuy.	*Z. J. Liu 4798*	CHINA	KJ733420	KJ733577	KJ733499	KJ733657	—
*G. setosus* Aver. & Vuong	*ZJY304*	Vietnam	OQ566816	OQ575581	OQ575610	OQ575553	—
*G. sinensis* Z.H.Tsi	*ZJY228*	CHINA: Zhejiang (Hangzhou)	OM985813	OK042953	OK172399	OK172401	OQ575646
*G. sinensis* Z.H.Tsi	*S1109*	CHINA: Sichuan (Wenchuan)	OP348890	OP373115	OP373120	OP373129	OP373125
*G. somai* (Hayata) Hayata	*Q. Liu 36*	CHINA: Fujian (Pingnan)	MK357128	—	MK357180	—	MK357220
*G. somai* (Hayata) Hayata	*Li 073*	—	—	MN124436	MN124436	MN124436	MN124436
*G. sororius* Schltr.	—	—	ERR7622420	ERR7622420	ERR7622420	ERR7622420	
*G. wenshanensis* Q.Liu, D.P.Ye & X.H.Jin	*ZJY292*	CHINA: Yunnan (Maguan)	OQ566820	OQ575586	OQ575615	OQ575558	OQ575642
*G. sumartranus* J.J.Sm.	*ZJY280*	Vietnam	OQ566818	OQ575583	OQ575612	OQ575555	OQ575639
*G. sumartranus* J.J.Sm.	*ZJY294*	Vietnam	OQ566819	OQ575584	OQ575613	OQ575556	OQ575640
*G. tianbaoensis* Q.Liu & Y.H.Tan	*ZJY275*	CHINA: Yunnan (Malipo)	—	OQ575585	OQ575614	OQ575557	OQ575641
*G. tianbaoensis* Q.Liu & Y.H.Tan	*Q. Liu 63*	CHINA: Yunnan (Malipo)	MK357120	MK357157	MK357186	MK357207	MK357214
*G. wolongensis* Jun Y.Zhang, B.Xu & Yue H.Cheng	*ZJY240*	CHINA: Sichuan (Wenchuan)	OM985810	OK172400	OK172402	OK172404	OK172403
*G. wolongensis* Jun Y.Zhang, B.Xu & Yue H.Cheng	*S1108*	CHINA: Sichuan (Wenchuan)	OM985811	OM974209	OM974211	OM974210	OQ575647
*G. xuanenensis* Z.H.Tsi	*ZJY298*	CHINA: Guizhou	—	OQ575587	OQ575616	OQ575559	OQ575643
*G. yunlongensis* W.H.Rao, L.J.Chen & Z.J.Liu	*ZJY301*	CHINA: Yunnan	—	OQ575588	OQ575617	OQ575560	OQ575644
*G. yunnanensis* Schltr.	*Q. Liu 60*	CHINA: Yunnan (Mengsong)	MK165469	MK357158	MK357185	—	MK357212
*G. yunnanensis* Schltr.	*ZJY278*	CHINA: Yunnan	OQ566821	OQ575589	OQ575618	OQ575561	OQ575645
*G. zhenyuanensis* Q.Liu & D.P.Ye	*Q. Liu 61*	CHINA: Yunnan (Zhenyuan)	MK357127	MK357146	MK357168	MK357199	MK357237
*Holcoglossum amesianum* (Rchb. F.) Christenson	*X.H. Jin 004/006*	CHINA	HQ404389	JF763779	HQ404439	—	—
*H. kimballianum* (Rchb. F.) Garay	*X.H. Jin 017*	CHINA	HQ452901	JF763787	HQ404452	—	—
*Luisia magniflora* Z.H.Tsi et S.C.Chen	*Z. J. Liu 3444*	CHINA	KJ733426	KJ733583	KJ733505	—	—
*Pomatocalpa diffusum* Breda	*TBG145837*	INDONESIA	AB217576	AB217752	—	EF670432	—
*P. spicatum* Breda	*Z. J. Liu 4589*	CHINA	KJ733438	KJ733595	KJ733518	KJ733675	—
*Saccolabium pusillum* Blume	*TBG144220*	INDIA	AB217580	AB217756	—	—	—

## References

[B1] Beentje H (2016) The Kew plant glossary: an illustrated dictionary of plant terms, 2^nd^ edn. Kew Publishing, Kew, 184 pp.

[B2] Chen XQ Tsi ZH Wood JJ (2009) *Gastrochilus* D. Don. In: Wu ZY Raven PH Hong DY (Eds) Flora of China, vol.25. Science Press, Beijing & Missouri Botanical Garden Press, St. Louis, 491–498.

[B3] Chen WS Lei M Ma CB Jin XH Wang XL (2022) *Gastrochilus xizangensis* (Aeridinae, Vandeae, Orchidaceae), a new species from Xizang, China. Phytotaxa 566: 219–226. 10.11646/phytotaxa.566.2.6

[B4] Don D (1825) Prodromus Florae Nepalensis. J. Gale, London, 256 pp.

[B5] Govaerts R Campacci MA Baptista DH Baptista PJ George A Kreutz K Wood JJ (2021) World Checklist of Orchidaceae. The Board of Trustees of the Royal Botanic Gardens, Kew. http://www.kew.org/wcsp/monocots/ [Accessed 13 August 2021]

[B6] Katoh K Standley DM (2013) MAFFT multiple sequence alignment software version 7: Improvements in performance and usability. Molecular Biology and Evolution 30: 772–780. 10.1093/molbev/mst010 23329690 PMC3603318

[B7] Lee CT Wu JH Wang YQ Hsieh SI (2023) *Gastrochilus yehii* sp. nov. (Orchidaceae) from Taiwan. Phytotaxa 587: 053–058. 10.11646/phytotaxa.587.1.7

[B8] Liao M Cheng YH Zhang JY Feng Y Liu GY Ye P Jin SL Lin HQ Xu B (2022) *Gastrochilus heminii* (Orchidaceae, Epidendroideae), a new species from Sichuan, China, based on molecular and morphological data. PhytoKeys 215: 95–106. 10.3897/phytokeys.215.91061 36761093 PMC9836483

[B9] Liu Q Song Y Jin XH Gao JY (2019) Phylogenetic relationships of *Gastrochilus* (Orchidaceae) based on nuclear and plastid DNA data. Botanical Journal of the Linnean Society 189: 228–243. 10.1093/botlinnean/boy084

[B10] Liu Q Wu XF Zhou SS Li JW Jin XH (2023) New species and record of *Gastrochilus* (Orchidaceae, Aeridinae) from China and Laos. Phytotaxa 585: 210–224. 10.11646/phytotaxa.585.3.3

[B11] Nguyen LT Schmidt HA Von-Haeseler A Minh BQ (2014) IQ-TREE: A fast and effective stochastic algorithm for estimating Maximum-Likelihood phylogenies. Molecular Biology and Evolution 32: 268–274. 10.1093/molbev/msu300 25371430 PMC4271533

[B12] Nguyen VC Averyanov LV Maisak TV Nguyen TLT Nguyen VK Truong BV (2022) *Gastrochilus pankajkumarii*, (Aeridinae, Epidendroideae, Orchidaceae) a new lithophytic orchid from southern Vietnam. Taiwania 67: 35–39. 10.6165/tai.2022.67.35

[B13] Posada D (2008) jModelTest: Phylogenetic model averaging. Molecular Biology and Evolution 25: 1253–1256. 10.1093/molbev/msn083 18397919

[B14] Pridgeon AM Cribb PJ Chase MW Rasmussen FN (2014) Genera orchidacearum: Epidendroideae, volume 6, Part 3. Oxford University Press, Oxford, 544 pp.

[B15] Rao WH Liu ZJ Zhang GQ Chen XH Huang J Chen GZ Chen LJ (2019) A new epiphytic species of *Gastrochilus* (Orchidaceae: Epidendroideae) from Yunnan, China. Phytotaxa 413: 296–300. 10.11646/phytotaxa.413.4.5

[B16] Ronquist F Huelsenbeck JP (2003) MrBayes 3: Bayesian phylogenetic inference under mixed models. Bioinformatics (Oxford, England) 19: 1572–1574. 10.1093/bioinformatics/btg180 12912839

[B17] Tang Z Wang Z Zheng C Fang J (2006) Biodiversity in China’s mountains. Frontiers in Ecology and the Environment 4: 347–352. 10.1890/1540-9295(2006)004[0347:BICM]2.0.CO;2

[B18] Thiers B (2025) Index Herbariorum: a global directory of public herbaria and associated staff. New York Botanical Garden’s Virtual Herbarium. http://sweetgum.nybg.org/science/ih

[B19] Tsi ZH (1999) *Gastrochilus* D. Don. In: Tsi ZH (Ed.) Flora Reipublicae Popularis Sinicae, vol.19. Science Press, Beijing, 399–420.

[B20] Tsi ZH (1996) A preliminary revision of *Gastrochilus* (Orchidaceae). Guihaia 16: 123–154.

[B21] Wu XF Ye DP Pan B Lin XQ Jiang H Liu Q (2019) Validation of *Gastrochilus prionophyllus* (Vandeae, Orchidaceae), a new species from Yunnan Province, China. PhytoKeys 130: 161–169. 10.3897/phytokeys.130.34555 31534404 PMC6728340

[B22] Xie JM Chen YR Cai GJ Cai RL Hu Z Wang H (2023) Tree visualization by one Table (tvBOT): A web application for visualizing, modifying and annotating phylogenetic trees. Nucleic Acids Research 51: W587–W592. 10.1093/nar/gkad359 PMC10320113 37144476

[B23] Xiong C Fu XY Tan K Huang Y Zhang HJ Wang YC Yang YB Yi SR (2025) *Gastrochilus obovatifolius* (Orchidaceae, Aeridinae), a new species from the Daba Mountains of Chongqing, China. PhytoKeys 252: 25–40. 10.3897/phytokeys.252.133501 39957784 PMC11829196

[B24] Ya JD Wang WT Liu YL Jiang H Han ZD Zhang T Huang H Cai J Li DZ (2023) Five new and noteworthy species of Epidendroideae (Orchidaceae) from southwestern China based on morphological and phylogenetic evidence. PhytoKeys 235: 211–236. 10.3897/phytokeys.235.111230 38033625 PMC10682981

[B25] Zhang JY Cheng YH Liao M Jin SL Qu CM Tan YC Plenković-Moraj A Xu B (2022) *Gastrochilus wolongensis* (Orchidaceae): A new species from Sichuan, China, based on molecular and morphological data. Ecosystem Health and Sustainability 8(1): 2101546. 10.1080/20964129.2022.2101546

[B26] Zhang JY Cheng YH Liao M Feng Y Jin SL He TM He H Xu B (2024a) A new infrageneric classification of *Gastrochilus* (Orchidaceae: Epidendroideae) based on molecular and morphological data. Plant Diversity 46: 435–447. 10.1016/j.pld.2023.08.001 39280969 PMC11390603

[B27] Zhang JY Cheng YH Liao M Jin SL Lin HQ Yang PY He H Xu B (2024b) *Gastrochilus balangshanensis* (Orchidaceae, Aeridinae), a new subalpine epiphytic orchid from the Mountains of Southwest China. PhytoKeys 247: 123–135. 10.3897/phytokeys.247.130755 39429518 PMC11489711

[B28] Zhou ZH Shi RH Zhang Y Jin XH (2021) Orchid diversity in China: Recent discoveries. Plant Diversity 43(5): 341–342. 10.1016/j.pld.2021.07.004 34816059 PMC8591207

[B29] Zhou CY Zeng MY Wu YW Li MH (2024) *Gastorchilus pseudodresslerii* (Orchidaceae; Vandeae), a new species from China: evidence from morphological and DNA analysis. Phytotaxa 634: 071–078. 10.11646/phytotaxa.634.1.6

